# Health Tract, No. 1

**Published:** 1865

**Authors:** 


HALL'S JOURNAL OF HEALTH. JANUARY. No. 1. VOL. 12. 1865.

### HEALTH TRACT, No. 1.

### INCONSIDERATIONS.

It is inconsiderate to eat when you don't feel like it. Sleepless nature calls for food when it is needed.

It is inconsiderate to eat to "make it even," to swallow a thing, not because you want it, but because you do not want it wasted by being left on the plate, and thrown into a slop-tub; but then it would have gone to fattening the pigs or feeding the cows, whereas it goes into your stomach when not needed, only to gorge and oppress and sicken.

It is inconsiderate to enter a public vehicle, and open a window or door without the express permission of each of the several persons nearest.

It is inconsiderate to ask persons nearest to a window or door of a public conveyance to open the same, for you thereby tax their courtesy to grant a request for your gratification, at the expense of their own preferences, and thus show yourself to have the selfishness of a little mind, and the manners of a boor; for you have no claim on the self-denial of a stranger, nor should you put such to the risk of injury to health for your mere gratification. The most that can happen from a too close vehicle is a fainting fit, which kills nobody, and which would rectify itself in five minutes if simply let alone; but an open window in a conveyance has originated pleurisies, inflammation of the lungs, sore throat, colds, peritonital inflammations, and the like, which have hurried multitudes from health to the grave within a week. The openness of a travelling conveyance has killed a hundred, where closeness has killed one.

It is inconsiderate to be waked up in the morning as a habit; it is an interference with nature, whose unerring instinct apportions the amount of sleep to the needs of the body, nor will she allow that habitual interference with impunity, under any circumstances.

It is inconsiderate to crowd the doors or vestibules of public assemblies, whether of worship or of pleasure; they are for purposes of ingress or egress, and to stand in them, to lounge or gaze about, to the incommoding of a dozen or more persons, within any five minutes, is not only impolite, but it is impertinent.

It is inconsiderate in passing out of a public assembly to stop an instant for purposes of salutation or conversation, to the detention of a dozen, or a hundred, or a thousand who are behind you.

It is inconsiderate to keep a caller waiting in a cold or dark or cheerless parlor for two, ten, or twenty minutes, to his risk of health or loss of time, merely for the purpose of showing a style of dress or personal adornment not habitual, or of making an impression of some kind foreign to the facts of the case.

It is inconsiderate to take a medicine, simply because it had cured some one else who had an ailment similar to your own. Of two donkeys on the verge of utter exhaustion and prostration, the one laden with salt was greatly refreshed, and had his burden largely lightened by swimming a river; the other with a sack of wool by the same operation doubled the weight of his load, and perished.

